# Deciphering the Potential of Probiotics in Vaccines

**DOI:** 10.3390/vaccines12070711

**Published:** 2024-06-26

**Authors:** Chang Xu, Amjad Islam Aqib, Mahreen Fatima, Sadia Muneer, Tean Zaheer, Song Peng, Essam H. Ibrahim, Kun Li

**Affiliations:** 1 MOE Joint International Research Laboratory of Animal Health and Food Safety, College of Veterinary Medicine, Nanjing Agricultural University, Nanjing 210095, China; 2 Department of Medicine, Cholistan University of Veterinary and Animal Sciences, Bahawalpur 63100, Pakistan; 3 Faculty of Biosciences, Cholistan University of Veterinary and Animal Sciences, Bahawalpur 63100, Pakistan; noormahreen63100@gmail.com; 4 Institute of Microbiology, University of Agriculture Faisalabad, Faisalabad 38000, Pakistan; 5 Department of Parasitology, University of Agriculture Faisalabad, Faisalabad 38000, Pakistan; teanzaheer942@gmail.com; 6 Institute of Traditional Chinese Veterinary Medicine, College of Veterinary Medicine, Nanjing Agricultural University, Nanjing 210095, China; payson@stu.njau.edu.cn; 7 Biology Department, Faculty of Science, King Khalid University, P.O. Box 9004, Abha 61413, Saudi Arabia

**Keywords:** probiotics, vaccine development, immune response, efficacy, safety

## Abstract

The demand for vaccines, particularly those prepared from non-conventional sources, is rising due to the emergence of drug resistance around the globe. Probiotic-based vaccines are a wise example of such vaccines which represent new horizons in the field of vaccinology in providing an enhanced and diversified immune response. The justification for incorporating probiotics into vaccines lies in the fact that that they hold the capacity to regulate immune function directly or indirectly by influencing the gastrointestinal microbiota and related pathways. Several animal-model-based studies have also highlighted the efficacy of these vaccines. The aim of this review is to collect and summarize the trends in the recent scientific literature regarding the role of probiotics in vaccines and vaccinology, along with their impact on target populations.

## 1. Introduction

The introduction of specific antigens into the body to stimulate and strengthen the adaptive immune system is known as vaccination. Vaccines are important for mitigating the severity of and preventing infectious illnesses [1]. Vaccine effectiveness (VE) can be measured using the disease incidence ratio between vaccinated and unvaccinated populations [2]. Many factors contribute to variations in VE across civilizations, including but not limited to economic, social, and biological factors [2]. The term “probiotic” refers in general to bacteria that have positive effects on the health of humans and animals, bearing the literal meaning “for life”. A probiotic is, technically speaking, a living microorganism that can provide health benefits beyond those that are inherent in food when consumed in sufficient quantities [3]. The effectiveness of microorganisms depends on their existence, abundance, and rapid growth. Specified amounts of each product should be indicated on labels to achieve the specified health benefits. Microorganisms such as *Lactobacillus* and *Bifidobacterium* are present in most probiotic products. Probiotics may be consumed as supplements, in dairy or non-dairy foods, or by ingestion [4]. The live microorganisms present in fermented foods are genetically identical to the strains found in probiotics. Consumption of probiotics can improve the health of the gut by regulating the microbiota therein, enhancing the immune system, improving the bioavailability of nutrients, reducing lactose intolerance symptoms, and reducing the risk of other diseases. Probiotics for companion animals provide numerous benefits, including immune system regulation, stress reduction, protection against intestinal pathogen-based ailments, and enhancement of growth performance [5]. Probiotic bacteria can serve dual roles in vaccination strategies. As vaccine candidates, they directly present antigens to the immune system, eliciting a targeted response. As adjuvants, they enhance the immunogenicity of co-administered vaccines, often by modulating the host’s immune response. For instance, *Lactobacillus casei* and *Bifidobacterium longum* have been used as adjuvants to improve seroconversion and seroprotecting rates in influenza vaccines. Similarly, *Bifidobacterium longum* BB536 has shown promise as a vaccine candidate by enhancing the production of IFN-γ, a cytokine critical for immune responses. This review paper examines probiotics, examples of probiotic strains and their impact on vaccine efficacy, and their future implications for the health of animals and humans.

## 2. Brief Background of Probiotics

Probiotics were first conceptualized by Metchnikoff in the early 20th century, and as part of his initial understanding, he stated that fermented milk containing beneficial bacteria might contribute to the longevity of Bulgarian peasants [6]. Probiotics as defined by WHO (the World Health Organization) and FAO (Food and Agriculture Organization) are “live microorganisms that, when given in adequate amounts, confer a health benefit to the host” [7]. Hill et al. [6] characterized probiotics in 2014 as specific components for which viable counts should be maintained until expiration and for which evidence supporting their health benefits is a necessity. Additionally, they stressed the importance of certifying all probiotics as safe for their intended use. Abd El-Gawad et al. [8] demonstrated that probiotic bacteria confer health benefits to humans and animals when administered in sufficient amounts. Probiotic microbial strains, generally regarded as safe, are delivered to consumers through yogurt or fermented milk [9,10].

The potential of probiotics has been reported for numerous species. Although some species may not be closely related, researchers in microbial taxonomy have grouped over 250 diverse bacteria under the genus *Lactobacillus* since 1901, when *Lactobacillus delbrueckii* was named the first *Lactobacillus* species [11]. In recent years, DNA sequencing and analysis have enabled experts to divide *Lactobacillus* into 25 genera, including an expanded *Lactobacillus* group, which incorporates organisms previously referred to as the *L. delbrueckii* group, as well as 23 novel genera [11]. The digestive tracts of healthy individuals and dairy sources are common reservoirs of *Bifidobacterium* and *Lactobacillus* species [12,13]. *Bacillus*, a Gram-positive bacterium capable of surviving in both aerobic and anaerobic environments, serves a crucial purpose in animal feed. Species such as *Bacillus subtilis* and *Bacillus licheniformis* are important probiotics in animal nutrition. *Bacillus* has several advantages due to its endospores, primarily its ability to be preserved in a dehydrated state at ambient temperature without compromising its viability [13]. The production of lactic acid as the primary product of carbohydrate fermentation is a key characteristic of *lactobacilli* [14]. *Lactobacillus*, with its diverse genome and physiology, is an excellent candidate for fermenting food, preserving foods for an extended shelf life, and improving health [15]. Its impact on the immune system, competitive exclusion properties, and high adhesion capabilities contribute to its safety for use [16].

## 3. The Impact of Probiotics on Enhancing Vaccine Effectiveness

**Figure 1:**
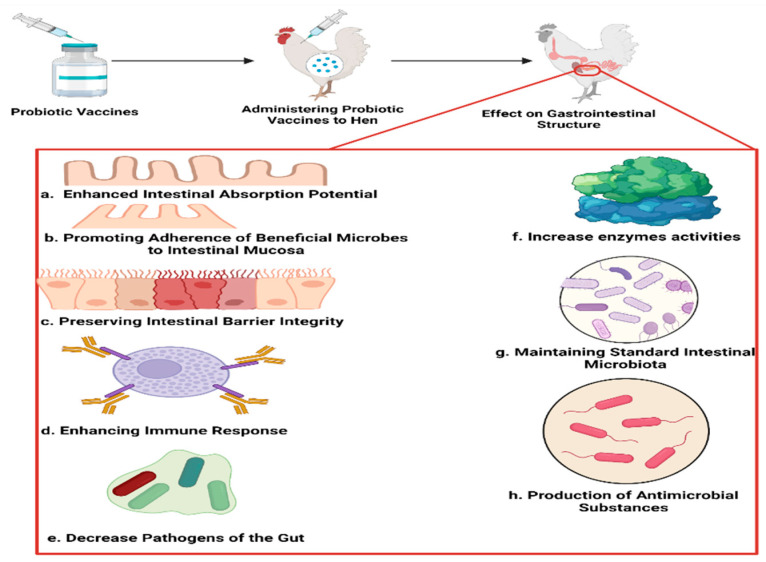
Impact of probiotics on intestinal health and immune system.

Probiotics have been found to influence both innate and adaptive immunity, leading to reducing incidences of illness and improving well-being [6], which also results in a reduced incidence of illnesses (Table 1). Lei et al. [17] conducted a meta-analysis showing that probiotics and prebiotics can enhance the response to flu shots. The analysis, involving 1979 individuals, manipulated seroconversion and seroprotection rates. *Bifidobacteria*, commonly referred to as BIF, are a category of beneficial and probiotic bacteria with numerous positive impacts on animal and human health. These effects include alleviating allergic symptoms, regulating lipid metabolism, and preventing infections. Makioka et al. [18] showed that *bifidobacteria* stimulate the host’s oral and systemic immune systems, resulting in increased interferon-γ (IFN-γ) levels, a cytokine associated with the T helper immune response, in babies given *B. longum* BB536. Additionally, Wu et al. [19] reported an increased proportion of IFN-γ-secreting cells relative to IL-4. Moreover, Soh et al. [20] provided evidence that administering a mixture of *Bifidobacterium longum* BL999 and *Lactobacillus rhamnosus* after hepatitis B vaccination could enhance antibody responses. Similar results were observed in individuals receiving seasonal influenza vaccines. Przemska-Kosicka et al. [21] demonstrated the potential to enhance total antibody titers and seroprotection by combining gluco-oligosaccharides with probiotics, including *B. longum infantis* CCUG 52486. The addition of *Lactobacillus paracasei (L. paracasei)* to influenza vaccination increased seroconversion rates, although no significant changes were observed in IFN, IL-2, or IL-10 concentrations. In their prospective study [22], they used a randomized placebo-controlled design with a double-blind method to investigate the potential effects of probiotics on vaccine efficacy. Specifically, they examined the effects of *Bifidobacterium infantis*, *Bifidobacterium bifidum*, *Bifidobacterium longum*, and *Lactobacillus acidophilus.* Nevertheless, statistical analyses yielded inadequate evidence to substantiate the claim that probiotics have an impact on the effectiveness of vaccination. The presence of *Bacillus licheniformis*, a facultative anaerobic bacterium, within poultry can enhance the rate of absorption and the surface area occupied by the gastrointestinal tract (GIT) (Figure 1). Moreover, it was found in another study [23] that probiotics stimulate the growth and multiplication of advantageous microorganisms, specifically *Bifidobacterium*, *Aspergillus awamori*, and *Lactobacillus* spp. Indeed, despite the efforts made, the introduction of this intervention did not yield any discernible impact on antibody generation following influenza vaccination, as noted by [24]. However, broiler chickens treated with probiotics containing specific strains such as *Lactobacillus acidophilus*, *Bacillus plantarum*, *Pediococcus pentosaceus*, *Saccharomyces cerevisiae*, *Bacillus subtilis*, and *Bacillus licheniformis* exhibited favorable outcomes across various parameters. The advantages observed in response to the SE vaccine included reduced fecal discharge and decreased re-isolation of *Salmonella Enteritidis* (SE) from vital organs like the liver, spleen, heart, and cecum, as well as a decrease in adverse effects associated with the live vaccine, leading to a decrease in mortality rates [25].

Different strains of probiotics have also been explored in connection with this subject matter. For instance, in a study conducted by Michael et al. [26], *Escherichia coli Nissle (EcN)* 1917 was utilized to establish colonization in Gn piglets previously subjected to antibiotic treatment, with their fecal microbiota then transplanted into human infants. These piglets were subsequently evaluated for immune reactions relevant to humans, primarily focusing on rotavirus (HRV). The findings indicated that EcN biofilms boosted both humoral and cellular immunity, along with an increase in the frequency of systemic memory and IgA+ B cells. Moreover, volunteers with dengue fever (DF) exhibited less severe symptoms after receiving *Lactococcus lactis* strains compared to the placebo group. Furthermore, in mouse models, probiotics were found to enhance the release of IFN-γ and TGF-β cytokines while reducing serum IgE and IL-4 cytokine concentrations [27]. Santos et al. [28] discovered that treatment with *Bacillus toyonensis* (*B. toyonensis*) strain BCT-7112 in conjunction with *Clostridium perfringens* epsilon toxin vaccination improved the humoral immune response in sheep, resulting in an increase in neutralizing antibodies. When lambs vaccinated with *Clostridium chauvoei* were exposed to *B. toyonensis* and *Saccharomyces boulardii*, there was a significant increase in antibody development and the expression of IFN-γ, Bcl6, and IL-2 genes. Additionally, *B. velezensis* notably reduced the levels of pigeon circovirus (PiCV) in pigeon spleens and feces while also stimulating Toll-like receptors 2 and 4 (TLR2 and TLR4). Fecal microbiome transplantation of *Saccharomyces boulardii* and *Clostridium butyricum* in pigs led to a significant increase in interleukins such as IL-22 and IL-17, which are effective against pathogens like *Mycoplasma hyopneumoniae* and porcine circovirus type 2 (PCV2). This therapeutic approach also resulted in a reduction in inflammation and oxidative-stress-induced damage, along with a decrease in intestinal barrier breakdown [28].

## 4. Probiotic-Based Vaccines in Animal Models

Probiotics, whether exogenous or endogenous, are bacterial species that interact with a variety of cellular components within the GIT, according to multiple pathways. The significance of intact and viable bacteria for expressing probiotic benefits cannot be overstated. *Lactobacillus* has been reported to positively impact vaccine efficacy (VE) in several studies, despite the contradicting observations reported in other studies. For instance, maternal supplementation of LGG *(Lactobacillus rhamnosus GG)* resulted in reduced specific antibody responses in infants vaccinated against tetanus, pneumococcal conjugate (PCV7), and *Haemophilus influenzae* type b [29]. Similarly, the consumption of probiotics containing *Lactobacillus* strains such as *L. paracasei* and *Lactobacillus casei* 431 led to a reduction in the duration of respiratory symptoms but did not show any significant effect on immunity to influenza vaccines [30]. In another study, a diet supplemented with *Lactobacillus parvairs* and MoLac-1 (heat-killed) probiotics produced similar results, indicating that these probiotics were unable to enhance the immune response post-vaccination [31]. Some of such studies have been summarized in Table 2.

### 4.1. Probiotics in Vaccines

**Figure 2:**
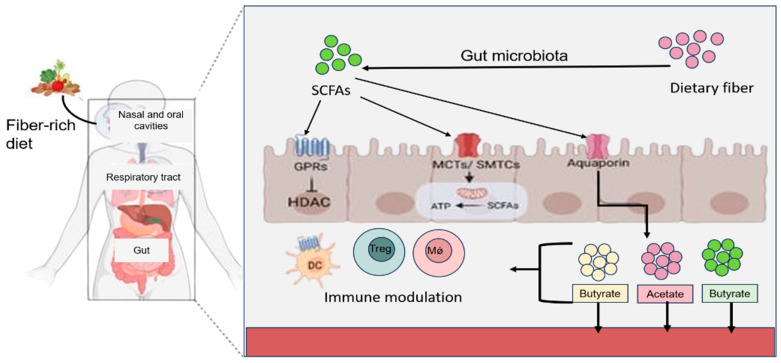
An illustration of production of short-chain fatty acids (SCFAs) by the gut microbiota. These metabolites interact with G-protein-coupled receptors (GPCRs) and monocarboxylate transporters (MCTs and SMTCs) to regulate gene transcription and energy production in intestinal epithelial cells (IECs). Moreover, SCFAs can traverse the intestinal mucosa, entering circulation to modulate both metabolic and immune functions. Key players in this process include dendritic cells (DCs), T regulatory cells (Tregs), and macrophages.

Probiotics are employed in animal vaccinations based on theoretical yet unverified mechanisms that could potentially impact immune function. Pathogen-associated molecular patterns (PAMPs) are transported to the gut-associated lymph nodes due to immediate alterations in the gut microbiota. This sets off signaling pathways necessary for the expression of various genes and the subsequent production of immune mediators, as PAMPs from probiotics interact with pattern recognition receptors (PRRs) to boost the host defense against pathogenic infections [40]. For instance, the ligands from *lactobacilli* bind to TLR2 in conjunction with TLR6, initiating cell signaling through MyD88 recruitment post TLR2 and TLR6 binding [41]. Transepithelial resistance is improved, and cytokine production is induced by this interaction [41,42]. TLR2 modulates immunological activity in some *Lactobacillus* strains by identifying peptidoglycan, a component of Gram-positive bacterial cell walls [43]. It has been demonstrated that *L. rhamnosus* and *L. plantarum* increase TLR2 expression in human cells (Caco-2) and that *L. casei* influences TLR expression in mice infected with *Salmonella* [44,45]. Furthermore, microbiological substances, specifically short-chain fatty acids (SCFAs), might be involved [45]. Short-chain fatty acids (SCFAs) have been shown in numerous studies to have a wide range of beneficial effects on the host’s gut and general health (Figure 2). These effects include anti-inflammatory qualities, the repression of NF-κB, which leads to the downregulation of IL1β, and the inhibition of histone deacetylases. Furthermore, SCFAs are essential for maintaining intestinal homeostasis. Several probiotics have regulatory qualities that may either directly or indirectly boost the production of SCFAs. Inflammation caused by *S typhimurium* can be reduced by *L acidophilus* in two ways: by increasing SCFA production directly or indirectly through its interaction with other SCFA-producing gut bacteria. In addition to balancing Salmonella-induced dysbiosis in mice, *L acidophilus* also increases bacterial resistance to diarrhea [46]. In an anaerobic environment, indigestible polysaccharides, including starch and inulin, cellulose, and pectin, can be fermented to generate SCFAs, which are small, organic carbon carboxylic acids with one to six carbon atoms. SCFAs such as formate can also be referred to as 2-methylbutanoate, 2-methylbutyrate, or valerate. The gut transports a variety of SCFAs into the bloodstream to produce significant impacts. According to Boets et al. [47], humans have systemic access to acetate, propionate, and butyric acid. The availability of SCFAs in the system allows them to function locally and systematically in the gut depending on their local and systemic effects. This protein also plays a key role in the binding of G-protein-coupled receptors (GPCRs), histone deacetylation, and modulation of immunity [48]. SCFAs are usually measured in fecal samples to determine the amount of SCFAs produced. According to McNeil et al. (1978) [49], the gut mucosa absorbs most of the SCFAs produced in the colon. SCFA levels may also be affected by gut epithelial integrity [50].

It is obvious that animal digestive tract cell types interact with probiotics and a variety of physiological outcomes occur depending upon the type and quantity of cells interacting. Salient actions include increased mucin formation, the stimulation of antimicrobial and heat shock protein synthesis, the restriction of dangerous organism development, the modulation of signaling pathways and cell survival in the intestinal epithelial cells (IECs), inhibition of the growth of harmful organisms, modulation of signaling pathways, and enhancement of cell survival [45]. Probiotics have demonstrated the enhanced production of defensive chemicals like defensins by IECs. Defensins, which are cysteine-rich cationic peptides, are used by the innate immune system to protect against pathogens. The intestine produces antimicrobial peptides from several cells, including epithelial cells, neutrophils, and Paneth cells. As it binds to its inhibitor IκB, the NF-κB protein stays inactive in the cytoplasm under normal circumstances. On the other hand, IKK phosphorylates IκB in response to pro-inflammatory stimuli, designating it for ubiquitination and subsequent proteasome destruction. By releasing NF-κB, this procedure enables it to enter the nucleus, where it attaches to promoters and starts transcription [51]. Probiotics use a variety of mechanisms to suppress NF-κB activation and regulate the release of downstream cytokines, a review by Thomas et al. (2010) [52]. For instance, direct contact with *Salmonella typhimurium PhoP* and *Salmonella pullorum* inhibits the production of IL-8 in polarized T84 epithelial cells by preventing IκBα from becoming polyubiquitinated and being degraded by the proteasome [51]. By producing reactive oxygen species, *Lactobacillus rhamnosus GG (LGG)* ATCC 53103 controls the breakdown of IκBα. The enzyme Ubc12, which neddylates the cullin-1 subunit of E3-SCFβ-TrCP, is deactivated by activated ROS. This inhibits the release of NF-κB by stopping E3 ligases from polyubiquitinating IκBα [53,54]. It is also pertinent to note that IκB degradation is also inhibited by other probiotic strains, which should be considered when developing any product.

In an epithelial cell model, *Lactobacillus rhamnosus* GG (LGG), both viable and heat-killed, decreased IκB degradation and the consequent translocation of NF-κB into the nucleus, leading to a reduction in TNF-induced IL-8 production [55]. Like this, pretreatment of epithelial cells with *lactobacillus casei DN-114 001* prevented IκBα degradation, which, in turn, reduced the NF-κB activation brought on by *Shigella flexneri*. The investigation of gene expression revealed that *L. casei* regulates several genes related to proteasomal and ubiquitination processes [56]. Additionally, IκB degradation is inhibited by other strains of probiotics. Ma et al. [57] discovered that by preventing transcription factors, preincubating live *Lactobacillus reuteri* with IECs suppresses the IL-8 expression that is induced by TNF and *Salmonella enterica serovar Typhimurium*. According to several investigations, *L. reuteri* can inhibit IκB degradation, which, in turn, prevents the TNF-dependent nuclear translocation of p65 [58]. Various probiotic species prevent NF-κB activation by inhibiting both proteasome and ubiquitination activities, while the exact mechanism is still unknown. One such organism that is well known for its proteasome-inhibiting properties is *Lactobacillus plantarum*. In an IEC model system, *L. plantarum*-conditioned medium suppresses the proteasome’s chymotrypsin-like activity, which stops IκB degradation. This limits both NF-κB binding activity and IκB degradation. *L. plantarum* inhibits proteasomes similarly to other inhibitors of proteasomes but without causing toxicity or cell death. The probiotic mixture VSL#3 suppresses proteasomal chymotrypsin-like activity without altering ubiquitination, according to Petrof et al. [58]. It has been observed in multiple studies that probiotics suppress the NF-κB pathway in IECs; however, their exact mechanism is unknown. For instance, secreted factors from *Lactobacillus fermentum DSMZ 20052* can diminish *Yersinia enterocolitica*-induced IL-8 secretion from IECs by suppressing NF-κB activation, concurrently reducing p38 MAPK activation, which also contributes to inhibition [59]. In an HT-29 cell model, *Bifidobacterium longum* and *Lactobacillus bulgaricus* could decrease NF-κB p65 nuclear translocation, leading to inhibition [60]. Furthermore, incubating *B. longum* with colonic biopsy explants from ulcerative colitis patients diminishes TNFα and IL-8 levels by inhibiting NF-κB. Supernatant from *Faecalibacterium prausnitzii DSM 17677*, a newly identified probiotic, is found able to suppresses IL-1β-induced NF-κB activation [61].

Heat shock proteins (hsps), which shield cells from damage and increase cell survival, are produced in greater amounts by stressed cells [62]. In the gut, almost half of the induced heat shock proteins, including HSP72 and HSP25, are expressed and help to preserve the barrier and tight connections between intestinal epithelial cells [63,64]. Tao et al. [63] found that actin is stabilized by hsp25, whereas cellular proteins are prevented from denaturing by hsp72. In the intestine, commensal or probiotic microorganisms promote the synthesis of cytoprotective heat shock proteins. In intestinal epithelial cells (IECs), *lactobacillus rhamnosus GG ATCC 53103 (LGG*)-conditioned media stimulates the production of HSP25 and HSP72. Heat shock transcription factor 1 and the mRNA levels of heat shock proteins 25 and 72 are increased because of LGG’s activation of p38 and JNK MAPKs. Blocking these MAPKs specifically inhibits the induction of hsp72 by LGG but not hsp25 induction [63]. HSP25 and HSP72 are also induced in cells fed with media conditioned with VSL#3, which contains three *Bifidobacterium* species and four *Lactobacillus* species, including LGG. According to Petrof et al., VSL#3 induces heat shock transcription factor 1, which regulates the expression of hsp genes in a similar manner to LGG. IECs are stimulated to produce hsp25 and hsp72 by *Bacteroides fragilis* ATCC 23745 through the phosphorylation of p38 and protein kinase B [64]. It may be required for these probiotic strains and host IECs to have a mutualistic interaction for the host to endure the stresses and insults that these strains inflict. As a result, when IECs are exposed to brief amounts of cell-free conditioned media consisting of *Lactobacillus rhamnosus GG (LGG)*, the transcription of hsp25 and hsp72 is increased.

### 4.2. Probiotic-Based Vaccine Response in Newborns

In one research study, the descendants of mice that received oral rotavirus (RVA) immunization in combination with Bifidobacterium breve YIT4064 displayed heightened protection against a subsequent RVA challenge compared to the descendants of mice solely inoculated with RVA [65]. These findings reflects a distinct link between probiotic intake and RVA-specific antibodies in the mother mice’s milk, feces, and intestinal contents after RVA vaccination. Suckling rats infected with the SA11 strain of RVA and those fed milk supplemented with *Lactobacillus casei* DN-114 001 showed enhanced small intestine brush-border enzyme activity and had reduced small intestine cellular vacuolization. The RVA levels in the entire intestines decreased, as happens with severe diarrhea [65].

*Bifidobacterium bifidum* supplementation in infants also increased RRV-specific IgA antibodies in their feces and blood. Pant et al. [65] found that *Lactobacillus rhamnosus* GG reduced diarrhea intensity and duration in BALB/c pups more than five other strains, which is also supported by the findings of previous studies conducted on newborns. Rotavirus-induced diarrhea was reduced, and local and systemic rotavirus-specific IgA responses increased, as found in a study supporting human therapeutic trials. The findings of this link may differ based on the type of probiotic strain. Novel probiotic strains like *B. longum subsp. infantis* CECT 7210 are being studied for RV-induced diarrhea in preclinical animal trials [65]. The impact of probiotic strains on animal hosts, including their growth performance and pathogen control, can vary significantly based on factors such as the specific antigen, supplementation circumstances, and the strain’s inherent characteristics. For instance, in animal studies, certain probiotic strains have been observed to enhance growth performance by promoting nutrient absorption and metabolism [66]. One example is the use of *Lactobacillus acidophilus* in broiler chickens, which has been shown to improve weight gain, the feed conversion ratio, and nutrient utilization, leading to an overall better growth performance. Conversely, other probiotic strains may exert their effects primarily through pathogen control mechanisms [67]. For instance, *Bacillus subtilis* has demonstrated efficacy in controlling pathogens such as *Clostridium perfringens* in poultry, reducing the incidence and severity of infections and thereby improving animal health and productivity. Additionally, the dosage of probiotic supplements and administration method can influence their effectiveness. In a study on pigs, it was found that higher doses of *Enterococcus faecium* resulted in improved growth performance and reduced diarrhea incidence compared to lower doses. Overall, the selection of probiotic strains for animal studies must consider not only their potential growth-promoting effects but also their ability to control pathogens relevant to the specific animal species and environmental conditions [68].

### 4.3. Probiotic-Based Vaccine Response in Adults

The relationship between probiotics and the immune response to vaccinations has been extensively studied. Most of the research, however, has problems due to variance in the probiotic strains, animal populations, and participant numbers [69]. Several mucosal vaccines have been tested in animal models, including the *Salmonella Typhi* Ty21a live-attenuated vaccine. In 2008, an attenuated trivalent influenza vaccine was administered through the intranasal route. The efficiency of this vaccine was determined through controlled research involving a small number of healthy animals administered with various probiotics. The findings of these tests were significant in terms of improvements in the prospective immune responses. According to the findings, different vaccine antigens and probiotic strains can result in varying degrees of antibody generation, specifically of IgA or IgG antibodies [69]. Paineau et al. [69] tested Dukoral, an oral cholera vaccine, on animals, with three groups as follows: *Bifidobacterium* (9), *Lactobacillus* (9), and *placebo* (20). After three weeks of feeding, the animals received two cholera vaccines one week apart. Animal research has shown that probiotic strains increase antibody production differently. None of the strains increased significantly in response to the cholera toxin [69].

## 5. Probiotics and Their Function in Different Vaccine Categories

There are significant benefits of using probiotics in vaccination strategies, such as improving the immune response to the antigen and reducing the risks associated with attenuated vaccines.

### 5.1. Probiotics Improve the Immune System’s Cellular and Humoral Responses

The probiotics have demonstrated ability to enhance the immune functions, Figure 2 summarizes brief interactions of probiotics within hosts. In a recent study, the probiotic administered-vaccinated group was able to successfully enhance both cellular and humoral immune responses compared to the control groups in a study [70].

***Humoral Immune Response:*** SPAP/S1 is a fusion protein formed by the N-terminal portion of the S1 subunit of the pertussis toxin expressed in mice, with a recombinant vector based on *Streptococcus gordoni* RJM4. In these animal models, researchers observed the presence of secretory IgA (SIGA) and immunoglobulin G (IgG) in the saliva, and recombinant proteins were noted to permanently colonize and remain within the mouth pulp [71]. Recently, a number of probiotics have been reported to affect sIgA levels in rodents and humans (Table 3). Overall, probiotic treatment led to increased production of sIgA, although the individual results varied. IgA-producing plasma cells was increased in a dose-dependent manner by *L. acidophilus*, *L. casei*, and yogurt [72]. Perdigon et al. [73] found that *L. casei* significantly increased the amount of antigen-specific IgA after *Salmonella typhimurium* inoculation. IgA secretion is sufficiently increased to prevent enteric infections. In a similar study, mice were pre-injected intraperitoneally with ovalbumin prior to consuming heat-killed *L. casei* and *Shirota*, and IgE production was assessed [74]. The reduction in serum IgE levels and the production of IgE after the intake of ovalbumin was attributed to *L. casei* and *Shirota*. In addition, *L.casei-* and *Shirota*-fed mice spleen cells produced significantly less IgE in response to ovalbumin stimulation than those from mice from the control group [74]. Despite the limited number of studies, *Lactobacillus* seems to be capable of enhancing IgA production in experimental animals.

The antigen that *Bacillus subtilis* can target and use to colonize the episomal expression system is the B subunit of a venom that changes shape when heated. Native toxins formed in enterotoxigenic *E. coli* strains in the laboratory can be detected and neutralized in mice that have been vaccinated and given modified *Bacillus subtilis* orally. *Bacillus subtilis* is also a way for *Clonorchis sinensis* antigens to move from one place to another [70]. 

### 5.2. Probiotics Enhance the Level of Antibodies

The probiotic *Escherichia coli Nissle* 1917 (EcN) was found to contain OspG and OspA protein antigens and Stx B subunits, which are useful for vaccines against ETEC strains. While this approach might have induced hormonal effects, it did not elicit specific antigen responses from T cells. However, EcN 1917 enhanced the anti-F4 and anti-F18 IgG antibody responses by generating various F4 or F4 and F18 fimbriae of ETEC [73]. Furthermore, *Lactococcus lactis*, bacteria typically present in food, were used to manufacture the *Helicobacter pylori* urease subunit B to assess the efficiency of an oral vaccine against *H. pylori* in mice. The results showed that vaccination provided excellent protection against Helicobacter stomach infection. A hybrid vaccination was created in another attempt to express the Transmissible Gastroenteritis Virus (TGEV) spike glycoprotein in *L. lactis*. Antibodies against TGEV spike glycoprotein and local immune responses in the mucosa were produced by mice in various experiments [75].

Probiotics are increasingly recognized for their role in enhancing the immune response, particularly in boosting antibody production. 

A hybrid vaccine was developed by expressing the TGEV spike glycoprotein in *L. lactis.* Mice vaccinated with this construct produced antibodies against the TGEV spike glycoprotein and exhibited strong local immune responses in the mucosa, indicating effective mucosal immunity and protection against TGEV [75]. *Bifidobacterium longum* was used to deliver rotavirus VP6 protein, a major inner capsid protein, to the gut. Mice vaccinated with *B. longum* expressing VP6 showed a significant increase in rotavirus-specific IgA in the intestines, offering enhanced protection against rotavirus infection [75]. This approach highlights the potential of probiotics to serve as live vectors for vaccines targeting enteric viruses. Probiotic and non-probiotic bacteria were found to express their potential to bind to norovirus genotypes GI.1 and GII.4 through VP1 capsid protein of norovirustein [76]. Mice immunized with LGG expressing VP1 exhibited enhanced production of norovirus-specific IgG and IgA antibodies, indicating strong systemic and mucosal immunity. This approach underscores the effectiveness of using LGG to boost immune responses against viral pathogens [77].

### 5.3. Probiotics Proliferate Immunocytes

In a study, mice were given the *L. lactis* PppA (LPA+) strain, resulting in the production of protective antibodies that shielded their lips and bodies against various strains of *Streptococcus pneumoniae*. Additionally, researchers utilized *L. limptis* to deliver the rotavirus spike protein particle VP8 in a mouse model [78]. This led to the generation of numerous IgA antibodies within the intestines of the vaccinated mice in vivo. Moreover, in vitro experiments demonstrated that serum from mice that were fed *L. lactis* with the cell-wall-bound RV VP8 antigen could completely thwart viral infection. Furthermore, eighty one-day-old hens received an oral vaccine comprising *Lactobacillus lactis*, capable of producing a synthetic fusion protein known as M1, derived from the H9N2 virus and the HA2 protein. Researchers observed the development of cytotoxic T cells and a humoral response after administering the probiotic-based vaccine to healthy female volunteers. The authors also developed codon-optimized human papillomavirus-16 E7 oncogenes using *L. lactis* [79]. In addition, a separate study examined *L. limptis*, engineered with human HPV E6 protein, and found significant increases in lymphocytes in the vaginal area, gut lining, and spleen following vaccination [79,80]. Indirectly and directly, probiotics have been found to influence the immune system. Among the indirect effects of cytokines are the enhancement of natural killer cells and macrophages, as well as the increased production of immunoglobulins. In addition to inhibiting the growth of other bacteria in the gut, they also boost the epithelial barrier of the gut and alter mucus secretion [80,81]. A decrease in anticommensal antibodies, innate cytokines, and Th17 responses was observed following oral gavage of *L. reuteri*, and as a consequence, immune hyperactivity may have been reduced. Heeney et al. [82] reported that *Lactobacillus* restores immunological homeostasis and immune function. According to Livingston and Hoffmann [82,83], *L. reuteri* promotes immunity in mice deprived of *Lactobacillus* spp. In the ileum and jejunum of this animal, *L. reuteri* produces pro-inflammatory cytokines and chemokines. Probiotics increase gene expression in the intestinal barrier, which increases tight junction signaling and strengthens tight junctions [84,85]. Actinin and occludin, as well as actin and ZO-1, can be phosphorylated to enhance physiological function or cytoskeletal structure. According to Walter et al. [85], *Lactobacillus acidophilus* inhibited HT29 and Caco-2 cell invasion and adhesion.

A multitude of vaccines have instituted precise standards for antibody titers, which function as metrics of individual protection. Although the significant achievements of vaccination programs are widely acknowledged [86], there is significant variance across people in terms of the amount of T cell and antibody responses elicited by immunization. For example, the range of neutralizing antibody titers and CD8+ effector T cell responses in humans after receiving the live attenuated yellow fever vaccine 17D, widely considered to be one of the best vaccines of its time [87], can vary by more than ten times between individuals. This observation implies that the immune responses of children exhibit a considerable level of variability. It has been shown that the cytokine responses to mycobacterial antigens in neonates who received the *Bacille de Calmette et Guérin* (BCG) vaccine vary considerably, by as much as a ten-fold difference [88]. Variations in immune responses induced by vaccines may give rise to discrepancies in vaccine efficacy, which may affect both the proportion of individuals protected and the duration of protection. It has been documented that the diphtheria, tetanus, and acellular pertussis (DTAp) vaccine has an 84% success rate in preventing typical disease and a 71% to 78% effectiveness rate in preventing milder symptoms of pertussis [89].

**Figure 3:**
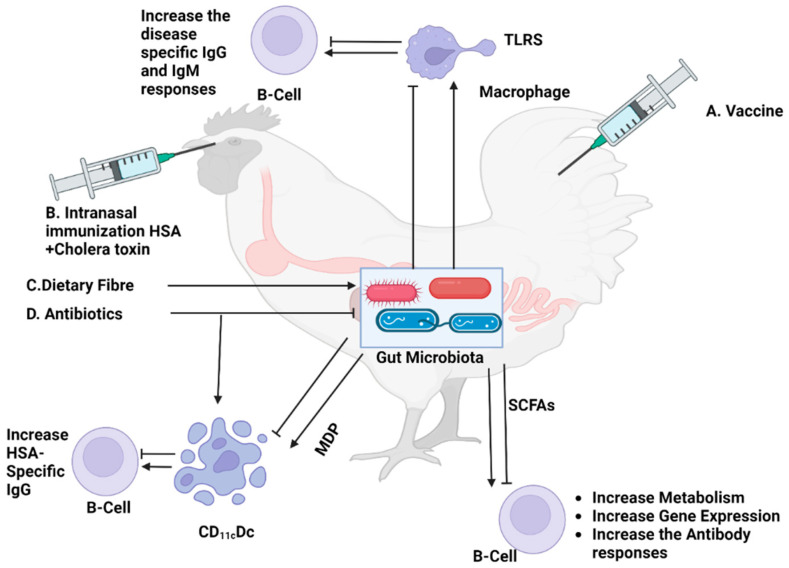
The mechanisms of effect of gut microbiota on antibody titer after vaccination. The gut microbiota play a crucial role in modulating the host’s immune system, impacting both innate and adaptive immune responses. After vaccination, certain bacterial communities in the gut can enhance antigen presentation and promote the maturation of dendritic cells. This leads to a more robust activation of B cells, resulting in higher antibody production. Additionally, microbial metabolites, such as short-chain fatty acids (SCFAs), can influence the differentiation and function of immune cells, further enhancing the immune response to vaccines. Dysbiosis, or an imbalance in gut microbiota, can impair these processes, potentially leading to suboptimal vaccine efficacy. Therefore, maintaining a healthy and balanced gut microbiota is essential for achieving optimal vaccine-induced immunity.

Conversely, there is considerable variability in the estimates of the BCG vaccine’s effectiveness in preventing pulmonary tuberculosis in infants, with the values varying from 0% to 80% [90]. Moreover, clinical studies have consistently observed that vaccines intended for populations in developing nations, including poliomyelitis and rotavirus vaccines, BCG vaccines, and others, demonstrate diminished levels of immunogenicity [89,91]. A multitude of factors, such as maternal immunization, genetic predisposition, and prior antigen exposure, have been recognized as possible contributors to the immunogenicity of vaccines. Nevertheless, recent studies suggest that the microbiota might also exert a significant influence on this phenomenon [89,91] (Figure 3).

Commensal bacteria are of considerable importance in sustaining the well-being of a wide array of animal species, encompassing quails, poultry, pigs, cows, sheep, horses, and other comparable organisms. This observed occurrence may be ascribed to the impact of bacterial metabolic residues, more precisely the short-chain fatty acids that are produced during the fermentation of resistant starch and dietary fiber. Acetate, propionate, and butyrate metabolites are of considerable importance in this particular process. The latter products play a crucial role in the regulation of energy balance, various physiological functions, and the modulation of digestive enzyme activity. Furthermore, research has shown that microbial metabolites can interact with the mucus system of the GIT, thereby influencing neurological diseases and intestinal homeostasis [92]. Metabolites produced by bacteria play crucial roles in several physiological processes within the host. A wide range of health and disease effects can be attributed to these metabolites, which are byproducts of bacterial metabolism. Intestinal epithelial cells produce short-chain fatty acids (SCFAs) such as acetate, propionate, and butyrate through the bacterial fermentation of dietary fiber in the gut [93]. These SCFAs function as energy sources, regulate immune responses, and maintain the intestinal barrier function. SCFAs can modulate inflammation and exert anti-inflammatory effects, thereby contributing to gut homeostasis. Host–microbe interactions and host physiology can also be influenced by bacterial metabolites such as indole derivatives. In addition to secondary bile acids and polyamines, other bacterial metabolites also play roles in host metabolism, immune function, and gut microbial composition [94].

### 5.4. Probiotics Increase the Production of Cytokines

*Lactobacillus casei* types are the most commonly used probiotics, and they are famous for their ability to boost the immune system. Mice and pigs had much stronger anti-PEDV IgA and IgG antibody reactions after being vaccinated with a genetically modified *L. casei* strain that expressed a dendritic-cell-targeting peptide [95,96]. These animals were given the vaccine to treat epidemic diarrhea in pig models. Interestingly, antibodies that were made against human papillomavirus 16 L2 in *L. casei* were also able to neutralize other kinds of human papillomavirus in a mouse model where the L2-specific antibodies were used to make the poly-glutamic acid synthase A protein. To protect the pigs from TGEV, recombinant *L. casei* vaccines were also given to them. It was noted that IL-17 was synthesized by mucosal and systemic immune cells, as well as a shift from Th1 to Th2 immune responses occurring. These procedures use a greater number of lactobacillus isolates. The administration of a genetically modified lactobacillus vaccine containing the pig rotavirus VP7 antigen enhanced the maturation of dendritic cells in Palmer’s patches, increased the levels of IFN- and IL-4 in the bloodstream, and promoted the release of B220+ B lymphocytes from lymph nodes in the mesenteric area. In murine experiments, it additionally elevated the concentrations of VP7-specific antibodies [96].

### 5.5. Probiotics and Cell-Mediated Immunity

Three-week-old chicks were administered genetically modified *Lactobacillus plantarum* bacteria capable of producing the H9N2 avian influenza virus. This virus was pathogen-free and capable of protecting cells and humoral immunity. When hens and mice were vaccinated with recombinant *L. plantarum* H9N2, they developed T cell immunity and high levels of antibodies that prevented hemagglutination. This virus, which contains a rabies-specific G protein, as well as a dendritic-cell-targeting peptide, boosted macrophage proliferation and induced a powerful Th1 immune response in mice. Recently, an edible vaccine was developed against SARS-CoV-2 using *L. plantarum* [95]. The spike gene is highly antigenic and acts well on the hybrid *L. plantarum* surface, according to the study’s findings. A strain of *L. plantarum* that expresses the SARS-CoV-2 spike protein was used to establish a new form of SARS-CoV-2 immunization, and a modified probiotic group was able to produce substantial levels of spike protein in the lab. *Lactobacillus pentosus*, which expresses the spike protein’s D antigenic domain, has been shown in mice to produce IgA and IgG antibodies against the transmissible gastroenteritis coronavirus (TGEV). It was also conceivable for vaccinated fish to produce antigen-specific IgM and KHV proteins capable of neutralizing the koi herpesvirus ORF81 protein [95]. The effects of *Streptococcus thermophilus* strains, commonly found in yogurt, on cytokine production were investigated using macrophage cell lines and T helper cell lines. Comparable effects were observed with active strains of *Lactobacillus bulgaricus*, *Bifidobacterium adolescentis*, and *Bifidobacterium bifidum.* The study revealed that heat-killed *S. thermophilus* affects various cytokines, including TNFα, IL-6, IL-2, and IL-5, in a strain- and dose-dependent manner. Notably, strains 133 and *C. alginicus,* of the *S. thermophilus* strains tested, significantly increased macrophage IL-6 production. Additionally, all four *S. thermophilus* strains strongly induced TNFα production. Moreover, IL-6 and, to a lesser extent, TNFα production were elevated when macrophages were co-stimulated with lipopolysaccharide (LPS) and cells from different lactic acid bacteria groups. Furthermore, IL-2 and IL-5 production was significantly increased after the simultaneous stimulation of T cells with phorbol 12-myristate-13-acetate [96]. Nicaise et al. [96] explored the impact of the bacterial flora on mouse peritoneal macrophage cytokine production. Tests were conducted with germ-free mice and mice implanted with either *E. coli* or *B. bifidum*. The mice implanted with bacteria exhibited significantly higher levels of IL-1 and IL-6 compared to the germ-free mice.

### 5.6. Probiotic-Based Bacterial Ghost Vaccination

#### 5.6.1. DNA Vaccines

Weak naked DNA vaccines often exhibit weak immunogenicity, which necessitates the use of adjuvants like alum to enhance the immune response. However, the current adjuvants available have several limitations, and there are limited choices for recombinant vaccine manufacturers. Nonetheless, the efficacy and immunogenicity of these vaccines can be improved through carrier transport [75]. Nucleic acid vectors are now widely used, including bacteria (e.g., *Bacillus Calmette-Guérin*), fungi (e.g., *Saccharomyces*), and viruses (e.g., *influenzavirus*, *adenovirus*, *poliovirus*). BGs (bacterial ghosts) can act as carriers and be internalized by various cells such as Caco-2 cells, HCDECs, DCs, and RAW 264.7 murine macrophages. In vitro studies have shown that 60% of macrophages (RAW 264.7) can express green fluorescent proteins after internalizing BGs containing reporter plasmids [97]. Additionally, DCs can internalize BGs and release IL-12, triggering Th1 immune responses. The uptake and activation of BGs by APCs offer a novel approach to immunization and in situ immunotherapy. BGs can carry plasmid DNA via the diffusion approach, where plasmid DNA diffuses into BGs through the lytic pores [77]. Furthermore, nucleic acids can non-specifically bind to BGs, with negatively charged DNA attaching to positively charged groups in the inner membrane, such as amines. Each BG can load 4000–5000 copies of plasmid DNA, ranging from medium to large plasmids [98]. These in vitro tests have demonstrated the feasibility of using BGs for nucleic acid transport.

Numerous studies have been conducted on BG-based nucleic acid vaccines in mouse models. These BG-based DNA vaccines have been shown to elicit stronger humoral (IgG upregulation) and cellular (Th1-type immune-related signal upregulation) immunity compared to naked DNA vaccines and BGs alone [99]. Jiao et al. [100] developed DNA vaccines using *Salmonella* ghosts to protect against *Neisseria gonorrhoeae*. They found that BG-based DNA vaccines induced stronger humoral responses (IgG upregulation) and lymphocyte proliferation than naked DNA vaccines and BGs alone. In a model experiment, Jiao et al. [101] used bone-marrow-derived DCs (BMDCs) to cultivate the vaccine. They observed that BG-based DNA vaccination enhanced DC maturation and activation (upregulation of cell surface costimulatory molecules CD80, CD86, CD40, and MHC-II) more effectively than naked DNA vaccines and BGs alone. These findings suggest that BG-loaded plasmid DNA vaccines have a stronger stimulatory effect on both humoral and cellular immunity compared to naked DNA vaccines. Cao et al. [102] prepared double-targeted DNA vaccines for the oral immunization of grass carp using DH5α ghosts coupled with plasmids harboring five exogenous fragments, including the gene encoding the invariant chain-like protein (Iclp) [100]. Endogenous *Iclp*-containing plasmids can efficiently navigate the MHC-II antigen presentation pathway. Double-targeted DNA vaccination significantly boosted the activities of intestinal mucus and serum in terms of three innate immune markers (SOD, LZM, and C3). These studies demonstrate that foreign genes can be derived from multiple plasmids or a single plasmid containing a fusion gene fragment and that antigenic genes in BGs can be internalized and expressed by APCs. Compared to naked DNA vaccines, the multigene BG vaccine induced stronger immune responses [101].

#### 5.6.2. Protein Antigen Vaccines

BGs serve two primary functions in the delivery of protein vaccines: (1) multi-epitope peptide BGs and (2) genetic engineering enabling the surface display of antigens and protein targets in BGs, enhancing their immunogenicity and targeting capabilities. Further research is needed to determine the optimal doses of proteins and BGs to elicit strong immune responses. In a study, chickens were protected against avian pathogenic *E. coli* APEC infection using BG-based recombinant vaccines containing ferrisiderophore receptors. Both the non-recombinant mixture and the recombinant BG vaccines significantly enhanced their IgG immune responses and reduced mortality rates due to infection. Notably, the recombinant BG vaccine was more effective in reducing mortality compared to the non-recombinant mixture [101]. In another instance, vaccines were developed against hand-foot-and-mouth disease by expressing the outer membrane protein A (OMPA) of *E. coli O157: H7*, along with antigenic proteins of *Enterovirus* 71 and *Coxsackie* virus. This vaccination induced mucosal immunity by boosting IgG and IgA production and protected mice from *E. coli* infection [103]. Additionally, Riedmann et al. created BGs by fusing the *Haemophilus influenzae* (NTHi) antigen (OMP26) into the S layer or the periplasmic space of *E. coli*, resulting in BGs that expressed protein antigens on their outer membrane and enhanced cellular immune responses [104]. BGs’ endomembranes, periplasmic space, and outer membrane proteins are inherently antigenic, capable of eliciting strong immune responses. Adjuvants containing BGs have been shown to significantly increase the immunogenicity of antigenic proteins compared to protein subunit vaccines [105]. The current research focuses on improving BGs’ protein-loading capacity. One approach involves expressing streptavidin in the inner membrane of BGs and biotinylating the target protein, facilitating immobilization through the biotin–streptavidin interaction. Genetic engineering techniques have also been employed to attach proteins to the bacterial membrane. For example, Sührer et al. [106] used galactosidase anchors to immobilize the enzyme cytochrome b5 on BGs [106]. In another approach, membrane vesicle fusion was used to seal BGs [107]. In summary, BGs’ structural components provide antigenic properties that elicit robust immunogenic responses. Adjuvants with BGs greatly enhance the effectiveness of antigenic proteins compared to traditional protein subunit vaccines [105,108].

## 6. Future Directions

Recently, there has been a significant rise in the use of probiotics in vaccines. As live organisms, probiotics are good for human health. They are used to avoid many health problems and are being studied for their ability to boost the immune system [109]. However, there are some issues to work out before probiotics can be used as an adjuvant for vaccines. A variety of probiotics are available, and each has its own advantages [110]. The addition of additives to vaccines, however, is not possible for every kind of vaccine. Probiotic bacteria may not be able to survive spray-drying because they are subjected to stressors like osmotic, oxidative, and mechanical stress and too much heat loss. If the water content drops below a certain level, cells may become dehydrated, which can kill beneficial microorganisms [111]. Most people think that probiotics are safe and may be good for your health, but there are not many studies that back up these claims. Some studies have found cases of severe infections in people with weak immune systems and premature babies. Probiotics are also used as adjuvants in vaccines, and they may facilitate dangerous bacteria to enter the bloodstream, which can cause serious infections and can have deleterious impacts on health and the immune system. Because of this, probiotics must be closely evaluated for their safety before they are used in vaccines [112]. There are a lot of probiotic products on the foreign market that are not drugs. They come in the form of dietary supplements, functional foods, natural health products, and other forms. Because of this, it is not clear where the regulatory system for probiotics fits in with other groups. Also, it is not clear how probiotics can be used as vaccine adjuvants and how they should be regulated. Labeling, quality, and safety standards for products can be inconsistent when there are not any rules in place. Since probiotics are not harmful, there may not be a robust immune system response when it comes across these bacteria. This means that probiotics cannot be used as adjuvants in all vaccines [113]. The pros and cons of probiotics and vaccines differ, but both can help prevent and treat diseases. Since probiotics affect animal health in a particular way, they have attracted more interest as a preventative measure [114]. Probiotics help the body to process food, use energy, and keep the immune system in check by colonizing the gut microbiota. They make the balance of microbiota in the gut better, lower the chance of infections, and boost the immune system [115]. Probiotic-based vaccines offer a promising avenue for future vaccine development. These vaccines use live probiotic bacteria as delivery vehicles for antigens, which can stimulate a targeted immune response [115]. By leveraging the natural gut colonization properties of probiotics, these vaccines can enhance mucosal immunity, providing protection at common entry points for pathogens [116]. Moreover, probiotic-based vaccines can be administered orally, which is more convenient and acceptable, especially for children and people with needle phobia [117]. Continued research and innovation in this field could lead to the development of more effective and accessible vaccines, improving global health outcomes [118].

## 7. Conclusions

Probiotics play a substantial role in vaccination strategies by augmenting immune responses and alleviating the potential adverse effects linked to specific vaccines. In addition to enhancing cytokine production, stimulating immunocytes, and augmenting cellular and humoral immunity, they function as efficacious vaccine adjuvants. Moreover, although probiotics may provide prospective health advantages and aid in the prevention of diseases through the enhancement of the immune system and the restoration of gut microbiota equilibrium, they ought not to be regarded as a substitute for vaccines. By eliciting targeted immunity and delivering durable protection against specific pathogens, vaccines remain crucial to disease prevention and treatment. Probiotics and vaccines possess distinct merits and demerits, and their use in preventing diseases and promoting general well-being may be complemented by one another. Further studies are required to explore potential technology that may couple probiotics and vaccines to enhance healthcare outcomes.

## Figures and Tables

**Table 1:** Salient studies on probiotics’ role as vaccine candidates and adjuvants.

Study	Probiotic Bacteria	Role	Vaccine Type	Key Findings
Lei et al. [17]	*Lactobacillus casei*, *Bifidobacterium longum*	Adjuvant	Influenza	Enhanced seroconversion and seroprotection rates in vaccinated individuals.
Soh et al. [20]	*Bifidobacterium longum BL999*, *Lactobacillus rhamnosus*	Adjuvant	Hepatitis B	Augmented antibody responses post-vaccination.
Przemska-Kosicka et al. [21]	*Bifidobacterium longum infantis*, *Lactobacillus paracasei*	Adjuvant	Seasonal influenza	Increased total antibody titers and seroprotection rates.
Makioka et al. [18]	*Bifidobacterium* species	Adjuvant	General	Stimulation of oral and systemic immune responses.
Wu et al. [19]	*Bifidobacterium longum* BB536	Candidate	General	Increased proportion of IFN-γ-secreting cells relative to IL-4.

**Table 2:** Salient studies on probiotic-based vaccines in animal models.

Probiotic Strain	Animal	Vaccine	Probiotics and Their Effects on the Response to Vaccines	Reference
*L. plantarum GUANKE (LPG)*	Mice	SARS-CoV-2 vaccine	Increased neutralization of SARS-CoV-2 antibodies within hours. SARS-CoV-2 vaccine increased specific neutralizing antibodies within 24 h.	[32]
*Lactobacillus* *plantarum Probio-88*	In vitro and in silico study	SARS-CoV-2 infection	In the spleen, MHC II expression on macrophages and B cells is elevated, the number of CD4+CD25+ T regulatory cells is reduced, IFN-α levels are higher at 21 dpi, and TGF-β4 expression is decreased.	[33]
*Lactobacillus*	Chickens	Herpes virus vaccine from turkeys	The findings reveal an upregulation of MHC II expression on macrophages and B cells within the spleen, accompanied by a decrease in the number of CD4+CD25+ T regulatory cells. Moreover, there is heightened expression of IFN-α at 21 days post-infection (dpi), coupled with a reduction in TGF-β4 expression.	[34]
*Bacillus velezensis*	Pigeons	Pigeon circovirus	There is a significant reduction in PiCV viral load in the feces and spleens of pigeons, along with up-regulation of IFN-γ, Mx1, STAT1, TLR2, and TLR4 gene expression.	[35]
*Lactococcus lactis NZ1330*	BALB/c Mouse Model	Allergy to Amaranthus retroflexus pollens	In addition to reducing serum IgE levels, enhanced Th1 and Treg responses are the best ways to improve allergies.	[36]
*L. acidophilus*; *L. plantarum*; *B. subtilis*; *B. licheniformis*	Broiler chickens	Salmonella Enteritidis vaccine	The detrimental impacts of the live vaccine on growth performance are mitigated, leading to a decrease in mortality rate, fecal shedding, and re-isolation of Salmonella Enteritidis (SE) from vital organs such as the liver, spleen, heart, and cecum.	[25]
*L.acidophilus W37*	Piglets	Salmonella Typhimurium strains	Vaccination efficacy doubled, correlating with a higher relative abundance of Prevotellaceae and a lower relative abundance of Lactobacillaceae in fecal samples. Additionally, an increase in the relative abundance of fecal lactobacilli was associated with firmer fecal consistency.	[37]
*Fecal microbiome+ Clostridium butyricum* and *Saccharomyces boulardii*	Gn piglets	-	The observed effects include increased plasma concentrations of IL-23, IL-17, and IL-22, alongside elevated levels of anti-M.hyo and anti-PCV2 antibodies. Moreover, there are reductions in inflammation and oxidative-stress-induced damage, coupled with enhancements in intestinal barrier function.	[38]
*B. toyonensis BCT-7112T*	Ewes of the Corriedale sheep	Recombinant Clostridium perfringens epsilon toxin	Several cytokines and transcription factors have been increased, including total IgG anti-rETX and isotypes IgG1 and IgG2, as well as Bcl6 mRNA.	[39]
*Saccharomyces boulardii*	Sheep	Clostridium chauvoei vaccine	There were 24- and 14-fold increases in total IgG levels, as well as specific IgG, IgG1, and IgG2 titers. Further transcription of IFNs, ILs, and Bcl6 mRNAs was observed.	[39]

**Table 3:** Effect of probiotics in modulation of humoral immunity in animals.

Probacteria	Species	Effect	References
*Lactobacillus casei Shirota*, oral (heat-killed)	Rodent	Inhibited splenocyte immunoglobulin (Ig)E production in vitro and reduced serum IgE levels	[71]
*L. casei*, oral (live)	Rodent	Increased secretory IgA (sIgA) levels and reduced incidence of enteric infections	[72]
*L. acidophilus* + *Peptostreptococcus*, oral (live)	Rodent	Reduced translocation and elevated levels of anti-E. coli IgM and IgE	[74]

## Data Availability

All the data are present in the manuscript.
